# Experimental Research on the Possibility of Changing the Adhesion of Epoxy Glue to Concrete

**DOI:** 10.3390/ma17225398

**Published:** 2024-11-05

**Authors:** Andrzej Szewczak, Grzegorz Łagód

**Affiliations:** 1 Faculty of Civil Engineering and Architecture, Lublin University of Technology, Nadbystrzycka 40, 20-618 Lublin, Poland; 2 Faculty of Environmental Engineering, Lublin University of Technology, Nadbystrzycka 40B, 20-618 Lublin, Poland

**Keywords:** adhesion, epoxy resin, microsilica, carbon nanotubes, sonication, cavitation, glue

## Abstract

Among the many methods of joining different materials, gluing is characterized by its most specific nature. In comparison with, for example, welded, screwed, or overlapped connections, a glued connection depends on the largest number of factors. Many of them are related to the phenomenon of adhesion, which is complicated by definition. It has many shapes and forms, and its existence determines not only the durability of such a joint but also the possibility of its execution. Epoxy polymers are among the most commonly used adhesives. Their extremely good parameters can be easily modified by additives in the form of fillers. Compatibility between the filler and the adhesive allows for further improving the adhesive parameters in the glued joint. However, in order to effectively combine the adhesive and the filler, different, often specific mixing methods must be used. The following study presents the results obtained in an experimental research program, the aim of which was to increase the adhesion of epoxy resin to a properly prepared concrete substrate. As a method to increase the final adhesion, the addition of microsilica and carbon nanotubes in an experimentally determined amount was selected. The use of sonication as a mixing method together with cavitation allowed for improving the parameters which determine the final adhesion of the adhesive to concrete. The parameters which were selected to describe the course of changes in the adhesion of the adhesive to the concrete substrate were the viscosity, free surface energy, surface parameters, adhesion, and SEM images of the tested resin in various modification configurations. The obtained results make it possible to form stronger and more durable adhesive joints during the reinforcement of concrete structural elements using fiber-reinforced polymer (FRP) composites.

## 1. Introduction

**Figure 1:**
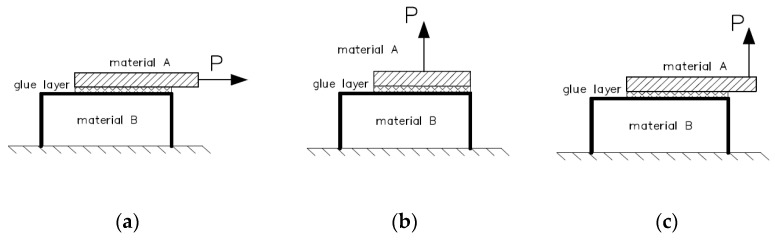
Stress states in glued joints: (**a**) shear, (**b**) peeling, and (**c**) tearing. P—force, arrow—direction of force application.

In bonding technology, the most important goal is to obtain a joint which effectively bears the expected loads. The theory considers several possible failure modes of bonded joints. Among the most commonly analyzed cases are the situations shown in Figure 1 [1,2,3,4].

**Figure 2:**
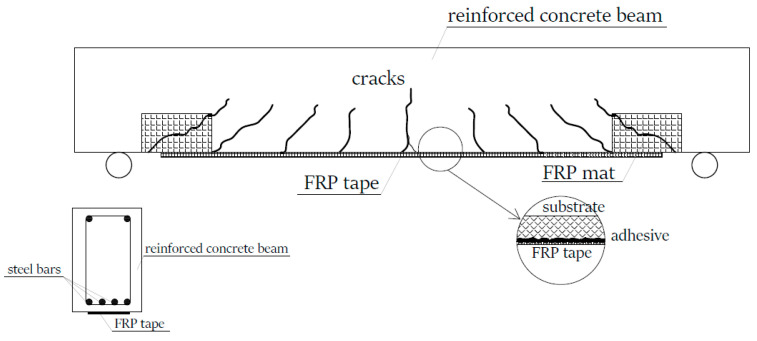
Diagram of the beam reinforced with FRP elements.

Although the most commonly analyzed state is the pure shear case, complex stress states are often encountered in such joints. Such a situation occurs, for example, in concrete and reinforced concrete structures reinforced with fiber-reinforced polymer (FRP) strips [5,6,7,8]. FRPs are produced as fibers (e.g., glass, carbon, aramid, or basalt) embedded in an appropriately prepared matrix in the form of a polymer, usually epoxy, polyester, or phenolic resin [9,10,11]. These composites, in the form of tapes, mats, rods, or meshes, are glued to the surfaces of various materials (i.e., concrete, wood, steel or ceramics) using adhesives, most often based on epoxy, polyester, or polyurethane resins. The adhesives used in these technologies are based on chain polymers, which are most often hardened with hardeners (chemically hardened). During the crosslinking reaction, it is possible to develop mechanical and adsorption adhesion between the adhesives, tape, and substrate [4,5]. Reinforcement is achieved by transferring the stresses acting in the reinforced element to the FRP element through the adhesive layer [6,7,8,9,11]. A diagram showing the operation principle of such reinforcement is presented in Figure 2.

As shown in the figure, FRP elements are placed in the zones of greatest stress. In the case of a reinforced concrete beam, this will be its bottom surface (reinforcement with a strap to level the effect of tension) and buttress zones (reinforcement of shear zones in the beam) [8,12,13]. As a result of the reinforcement, a part of the loads acting on the beam is carried by FRP elements. Their use is determined by a number of their highly favorable characteristics. First of all, these are low weight, extremely good strength, and durability [9,10,11]. A common feature of FRP composites is also their relatively low value of longitudinal strain, which is associated with low values for the elastic modulus. This means the practical absence of the range of plastic work (the so-called brittle material work). Therefore, the adhesive layer between the post surface of the reinforced element and the FRP composite plays a key role in assessing the effectiveness of such reinforcement [11,13].

The effectiveness of the beam reinforcement method shown in Figure 2 is determined by several aspects:

- Surface condition: The method of preparing the surface of concrete should take into account the bonding technique, such as by pressing the surface layers to be joined, the type of materials to be joined, their physical, mechanical, and chemical properties, as well as their near-surface structure and the method of proper treatment [1,14,15]. In the case of concrete, surface treatment methods (i.e., grinding, sandblasting, and shot peening) focus on the removal of weak layers composed of cement milk residue, dust, the formation of a developed surface, and in some cases, the exposure of aggregate grains located under a thin layer of grout on the surface of the concrete element.
- Bonding method: Ensuring adequate wetting by the adhesive largely depends on the condition of the surface and the ability of the adhesive to fill the non-uniformity of the substrate. The formation of a relative constant in terms of the thickness of the adhesive layer [14,16,17] promotes a favorable distribution of stresses, while the theory takes into account a great number of methods for their consideration and calculation. In the literature, one can find information about various thicknesses of adhesive joints used, which can range from a few to several atomic layers (a few nanometers), through layers of thickness from tens to hundreds of μm, and to layers typically used in technology in the thickness range of 0.05–0.15 mm. The last range results from a certain assumption saying that at an adhesive joint thickness <0.2 mm, linear stress distribution is possible [17]. Nevertheless, one can also find studies describing other, greater adhesive thicknesses [8,14].
- Type of glued materials: Construction materials are characterized by a unique surface structure which is reflected in the results of roughness tests, such as concrete or steel surfaces subjected to different surface treatments [1,17,18,19]. When designing bonded joints, the mechanical parameters of the bonded materials should also be taken into account, as they affect the course of the phenomenon during the transmission of stresses through the adhesive layer. In this case, the stiffness of the bonded materials, which depends on the strength, is of greatest importance [8,15,20].
- Type and preparation of the adhesive: As studies have shown [1,8,21,22], the strength of the bonded joint is determined primarily by the type of adhesive used and its chemical composition. The adhesive should be selected with a view of the expected and required adhesion to the substrate and durability. For the strengthening of structures and bonding of materials, hardening adhesives, chemically curable adhesives, and hybrid adhesives are used [1,22,23]. The analyses described in [1,22], among other works, proved that polymer adhesives subjected to crosslinking are characterized by higher durability, as shown in [1,22,23]. In [1,22], it was proven that polymer adhesives subjected to crosslinking are characterized by greater durability, stiffness, and resistance to chemical agents and creep than adhesives with chain structures. The type of curing agent affects the course and speed of the crosslinking reaction to a different extent, as shown in [24,25]. The crosslinking process can also be improved by using support networks, and the crosslinking process can also be improved through the use of interpenetrating polymer networks (IPNs), which are designed to further strengthen the joint by using curable and crosslinked copolymers to form interpenetrating structures (double crosslinking) and require the selection of appropriate mers [25,26] in the form of powders or fibers [27,28,29,30]. Their particles, appropriately mixed with the adhesive, can strengthen the adhesive layer (fibers) or form additional chemical bonds with the substrate, which improves the adhesion of the adhesive. In addition, fillers affect the performance, strength, and processing parameters. The list of fillers which can be used in the processing and production of polymers is constantly expanding, but the most commonly analyzed options for the addition of various organic and inorganic powdered phases are in the form of flours, powders such as chalk, mica, carbon black, microsilica, stone meal, brick meal, and talc. Some of them are added to polymers as only filler substances and cause a partial reduction in polymer wear (without a simultaneous negative influence on its properties), while another group of fillers enters into chemical reactions with polymer chains, thus forming a kind of composite and positively influencing the mechanical, processing, and other characteristics [27].
- Working conditions of the bonded joint: The correct determination of the loads which will affect the joint (static or dynamic), the resulting type of stress (tangential or normal stresses), and the nature of the loads (alternating, fatigue, or constant) is extremely important in the design of bonded joints [1,8,14,31,32,33]. In addition, in some cases, the so-called short-term and long-term strength is also determined, which is usually lower due to the weakening processes of the adhesive joint under the influence of fatigue, relaxation, aging, creep, and environmental factors, mainly UV rays and temperature [1,27,34].
- Shear strength: In the analysis of bonded joints, it is the leading parameter due to its high load-bearing capacity, although it is often not decisive. The shear stress value depends on the type of adhesive, the condition of the surfaces to be joined, the area of the contact area, the thickness of the adhesive joint, the temperature, and the physical and mechanical properties of the materials in the joint. It is assumed that shear stresses have the highest value at the edges of a weld [8,20,33]. In order to determine and describe the model which allows determining the stress values, numerous theories are used. Described in the literature [8,33,35,36] are theories, each of which contain some simplifying assumptions, such as the Volkersen, Goland and Reisner, Benson–Kesley, and Harth–Smith theories. These theories do not take into account the distribution of stresses over the thickness of the adhesive weld and are included in the group of linear elastic analyses. Moreover, these analyses neglected normal stresses from peeling, which in practice occurs quite often and complements the complex state of stress in the adhesive layer between the materials to be joined. More complex theories, such as those of Grimes and Greiman, take into account the so-called adhesive flow in the weld (often adhesive modified with additives in the form of fillers). Unfortunately, the two-dimensional nature of the analyses still does not allow all effects of loads in the adhesive layer to be taken into account [8]. Only the introduction of accurate three-dimensional analyses using computer software and FEM analyses allowed all types of stresses to be taken into account (In practice, it turned out that pure shear in adhesive joints occurs extremely rarely.) [8,37,38].

When analyzing specific cases of reinforcing concrete and reinforced concrete elements by bonding FRP composites, it should be assumed that adhesion to the bonded materials will be governed by the following [1,15,17,32]:

- Mechanical adhesion: This results from the mechanism of locking and interlocking of the adhesive layer against the irregularities of the substrate.
- Adsorptive adhesion: This is described as a series of chemical interactions and bonds formed between the molecules which make up the chemical composition of the adhesive and the surface of the materials to be joined. In practice, there is an exchange of electrons between the materials, as well as their sharing and the formation of permanent and temporary bonds. In this case, the Lewis theory, or the “theory of acids and bases”, complements the phenomenon of adsorption adhesion. According to its assumptions, in an adhesive joint, the acid (electron acceptor) is the adhesive with its modified structure, while the concrete substrate is the base (electron donor) [8,37,38,39].

The latest development described in [40,41], among other works, is the possibility of analyzing connections between materials by means of molecular dynamics. Advanced and complex computer analysis of intermolecular interactions, taking into account the actual movements of molecules between materials, allows analysis of phenomena of both predictable and probable natures, taking into account, for example, the complex state of stresses in a bonded joint subjected not only to mechanical loads but also environmental factors. These analyses make it possible to fully explain the physicochemical phenomena occurring in a solid phase, such as an adhesive.

In accordance with the assumptions presented above, a research program was carried out to analyze the effects of modification of epoxy adhesive with filler additives, namely microsilica and carbon nanotubes. The main purpose of the tests was to determine the possibility of increasing the adhesion figure of the adhesive used to glue carbon fiber reinforced polymer (CFRP) tapes to a concrete substrate modified by grinding. In order to fully describe the phenomena occurring between the adhesive, the concrete substrate, and the tape, the parameters of the ground surface and the adhesive were determined both in liquid form (density and viscosity) and after its crosslinking (surface free energy and adhesion), tested using the pull-off method. In addition, the adhesive was subjected to SEM analysis to determine changes in the structure of the adhesive after the addition of microsilica and nanotubes. The goal of the research was achieved, while the study explains the processes responsible for increasing adhesion. The subject matter undertaken is in line with contemporary research trends related to the search for methods for modifying different types of polymers with various fillers.

## 2. Materials and Methods

### 2.1. Components and Used Mixtures

#### 2.1.1. Epoxy Resin

The adhesive chosen for the study corresponds to Epidian 53 (Ciech Sarzyna, Nowa Sarzyna, Poland), the basic parameters of which are shown in Table 1. It is an epoxy paste without the addition of fillers, such as quartz flour or chalk. The glue underwent crosslinking after the addition of a hardener. In the described tests, the hardener used was Z-1 (triethletetramine) with a density of 0.98 g/cm^3^ (22 °C) and a viscosity of 20–30 mPa∙s. The hardener is designed to induce a crosslinking reaction in the epoxy resin and cause a transition from a liquid state to a glassy state.

#### 2.1.2. Fillers

In the study, two fillers in the form of powders were used as additives, aiming to increase the adhesion of the adhesive to the concrete substrate: micro-silica (BASF, Ludwigshafen, Germany) and carbon nanotubes (NANOCYL, Sambreville, Belgium). The basic characteristics of the fillers are shown in Table 2.

The Epidian 53 adhesive was modified by adding fillers in the amounts described in Table 3. The table also shows the formulations used in the research.

### 2.2. Methodology, Equipment, and Test Program

The research program included several successive research phases, according to the chronology of the research conducted.

#### 2.2.1. Preparation of the Adhesive

Sonication using a UP 400S stationary sonicator (Hielscher Ultrasonics Gmbh, Teltow, Germany) was performed as a method to mix the fillers we used [42,43]. In the case of powder fillers, ordinary mechanical mixing methods do not guarantee uniform dispersion and distribution of the filler molecules in a dense resin. Sonication, the main effect of which is cavitation, not only allows mixing of two phases but also chemical activation (chemical sonoreactions) [24,44] of the filler in an adhesive structure. In this particular case, a sonicator with a power of 400 W emitting ultrasonic waves at a frequency of 24 kHz was employed. The sonication time, which was determined experimentally, amounted to 3 min.

#### 2.2.2. Determination of Changes in Viscosity of the Adhesive

Viscosity and its changes allow determining the behavior of an adhesive on a target surface during its application [45]. As a quantity which defines the rheology of the resin, viscosity affects the process of covering the concrete with an adhesive and filling the irregularities which occur. In this study, the viscosity of the adhesive in the ER53 series (unmodified adhesive) was determined when the sonicator was turned off and at 22 °C (after the resins cooled). Measurements were carried out using a rotational viscometer of type H2 from FungiLab (FungiLab, Barcelona, Spain) at a spindle rotation speed of 60 rpm. As an auxiliary parameter for describing later test results, the temperature of the resins at the time of cessation of sonication was also determined.

**Figure 3:**
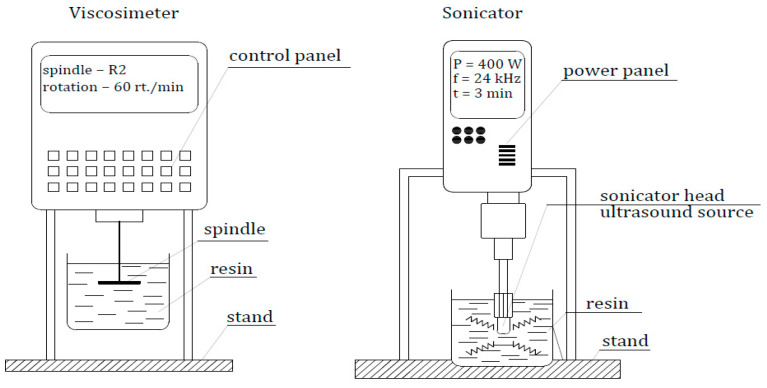
Scheme of the viscosity and sonication test stands with the test parameters. P = power; f = frequency; t = time.

Figure 3 shows a summary schematic of the stations for carrying out the sonication process and viscosity measurements.

#### 2.2.3. Determination of Surface Free Energy of Resins After Curing

**Figure 4:**
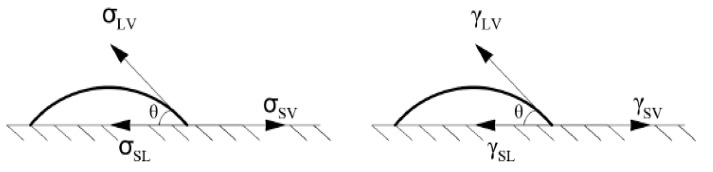
Distribution of surface tension forces and surface energy at the solid–liquid interface. σ = surface tension; γ = surface free energy; S = solid phase; L = liquid phase; V = gas phase. Double indices refer to the boundary of individual phases.

The surface free energy is a parameter which allows determining the contribution and changes of adhesive adhesion on a target application surface. In the described test program, the Owens–Wendt method [15,46,47] was chosen to describe the SFE. This method makes it possible to determine both the dispersion component, which refers to the behavior of the adhesive on the surface at the time of application, the physicochemical phenomena, its spreading ability and degree of dispersion, as well as the polar component, which is directly related to the adsorption phenomena at the adhesive–substrate interface and the exchange of electrons between molecules. This is possible due to the use of two types of measuring fluids (among many others mentioned in the literature) described in the literature: distilled water and diiodomethane. To determine the SFE, the angle between the surface of the sample and the line tangent, the shape of the droplet is determined first (Figure 4), and then the component values (dispersive and polar) and the total SFE value, which is the sum of the components, are calculated. Measurements were performed on the surfaces of samples made of resins of each series, which were 10 cm in diameter and 5 mm thick. For each series, 20 measurements were conducted (4 samples with 5 measurements each), and a PGX goniometer (Klima-test, Wroclaw, Poland) with a measurement accuracy of ±1° was employed.

#### 2.2.4. Measurement of Adhesion of Modified Resin to Concrete Substrate

**Figure 5:**
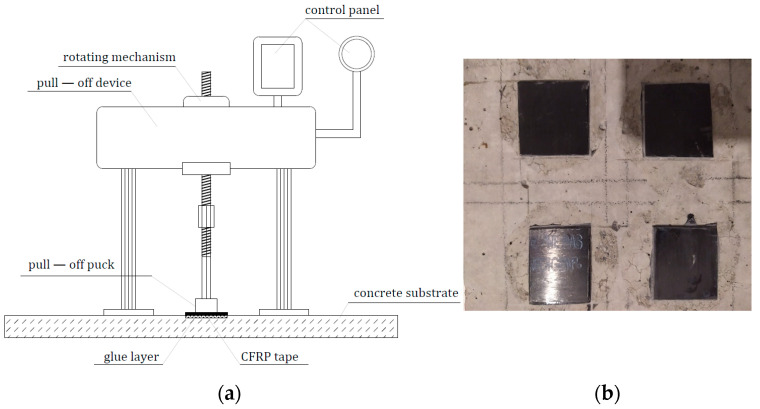
The pull-off research position (**a**) and the CFRP tape samples glued to a concrete surface (**b**).

The stage of the research which finally made it possible to determine changes (and any increase) in the adhesive adhesion to concrete was a series of tests using a modified pull-off method (Figure 5). For this purpose, the C30/37 concrete was first prepared to ensure that the pull-off strength of the concrete itself was at least 2 MPa. Then, after 250 days of storage, the surface of the specimens was ground to remove weak boundary layers and aggregate detachment (the concrete was made of granite aggregate with a 4–16 mm grain size, 0–2 mm sand, and CEM-II 42.5 R cement). After grinding, the specimens were dried, and the surface parameters were measured using a T8000 RC120-400 contact profilometer from Homme-Etamic (Charlotte, NC, USA). Final changes in the adhesion of adhesives to the concrete substrate were obtained by examining the changes in the pull-off adhesion of 2.5 × 3 cm sections of CFRP tape glued to the ground concrete surface with the adhesives shown in Table 3. The size of the CFRP tape sections was dictated by the measurement capabilities of the pull-off Dynatest device (Gainesville, FL, USA), with a load range of 0–25 kN. When testing each adhesive, 6 measurements were performed.

#### 2.2.5. Analysis of the Microstructures of the Resins Using the SEM Method

The final stage of the study was to find differences in the structures of cured resins subjected to the described modifications. This goal was achieved using a Quanta 250 FEG (FEI, Hillsboro, OR, USA) scanning electron microscope (SEM). The built-in control modules of the microscope allow a highly accurate picture of the structure of the material under study. During the study, the accuracy and precision of mounting the samples under the microscope was extremely important. The analysis was carried out under a high vacuum, while the samples themselves were attached with carbon tape to aluminum holders.

## 3. Research Results and Discussion

### 3.1. Viscosity and Density Results

**Figure 6:**
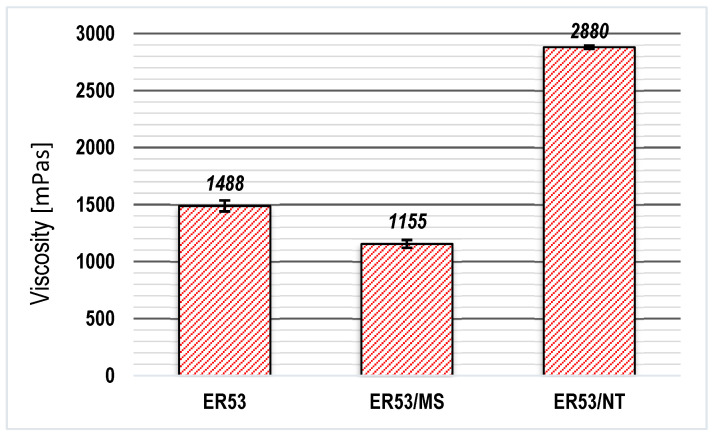
Results of viscosity tests.

The graph presented in Figure 6 shows the changes in viscosity of the tested resin subjected to two types of modification. The applied amounts of fillers—0.5% in the case of microsilica and 0.1% in the case of carbon nanotubes—did not cause changes in the density of the adhesive.

**Figure 7:**
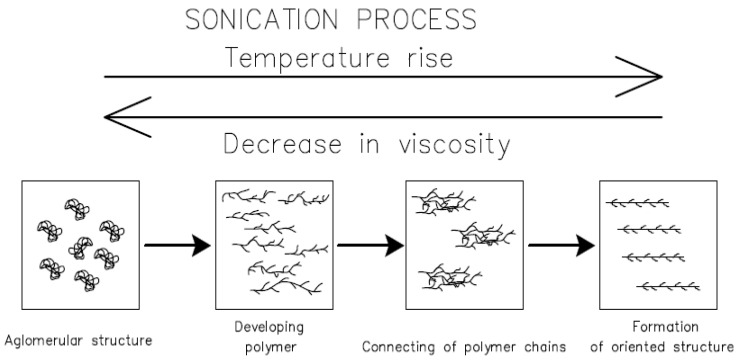
The effect of sonication on the polymer structure.

However, the modification method adopted caused significant changes in the viscosity values. Viscosity, as a phenomenon related to the cohesion of the molecules of the adhesive in a liquid state, largely depends on the distribution of mers in the polymer chains, as described in [45,48,49]. The peculiar structure of epoxy resins, having so-called epoxy groups, allows forcing a change in the locations of the groups under the action of a strong medium emitting high energy. In the case of the ER53/MS series, a 22.4% decrease in viscosity was found, but the ER53/NT series showed a marked increase of more than 93.5%. Thus, it can be seen that the type of filler used is crucial. A careful analysis of the existing relationship between the adhesive, filler, and sonication process brings about concrete conclusions. The reason for this is directly due to the phenomena occurring during the sonication process, which was also described in other works [48,49,50,51,52], and this is also shown in the diagram in Figure 7. Sonication is a dynamic, step-by-step process and results in certain important characteristics. Ultrasonic waves, propagating in an initially dense medium such as resin, are not able to immediately contribute to the effect of mixing the filler with the adhesive. The course of sonication therefore consists of certain stages, the key effect of which is the formation of the phenomenon of ultrasonic cavitation [49,50,53,54].

Initially, the high frequency of the waves caused a gradual increase in temperature and, consequently, a decrease in viscosity. At the same time, the initial agglomerates are composed of chemical polymer molecules, which in the resting state of the adhesive assume the shape of clusters, undergo unfolding, straightening [48]. This process also increases their chemical activity. Further impacts of cavitation, due to local changes in pressure and temperature, cause the formation of cavitation bubbles, which in the case of polymers are composed of gases such as air, oxygen, nitrogen, and volatile hydrocarbons. Their formation and collapse are extremely fast but still enough to affect the course of bonding of polymer chains to filler molecules [52,53,54]. The higher the temperature, the more intense the vibration of molecules becomes from both polymers and fillers. Some of the mers undergo reciprocal relocation, and between the chains themselves, voids can then form, into which filler molecules can enter by penetration. However, the important factor is their nature, which affects the process of bonding to the polymer structure, and consequently, the state of rheology of the polymer is modified. This is because with the movement of molecules, electrons move, which are responsible for the formation of permanent or temporary chemical bonds. During sonication, any implosion of a cavitation bubble allows conditions for radical polymerization to occur [24,51,55]. Permanent and temporary chemical bonds, characterized by different energy and durability, in polymers lead to an altered configuration of the main and side polymer chains. Each of the resin series studied was characterized by a pronounced increase in temperature, which was the result of vibration and friction of the chemical molecules in their original composition. For this reason, even sonication alone can be a way to modify a polymer [24]. After sonication stops, the temperature of the polymer drops again, and the viscosity increases. During this time, the structure of the polymer stabilizes. However, it reaches a new, altered state resulting from the altered location of the mers and other molecules to be associated with the polymer chains.

The series with the addition of microsilica was characterized by a decrease in viscosity. The presence of a filler with clearly spherical particles containing silicon atoms (microsilica), even in a relatively small amount, changed the course of reorganization of the polymer structure [55,56]. The microsilica particles may have been the seeds of the cavitation centers initiating the changes during sonication. Since the main component of microsilica is silicon, which is an element belonging to group IV, the main group of the periodic table, it has as many valence atoms as the main component of epoxy resin: carbon. Under the right conditions induced by sonication, this can allow the formation of temporary but relatively permanent coordination and hydrogen bonds, which are mainly related to the van der Waals bonds described in numerous works. Molecules excited during sonication can form numerous new structures which determine further parameters of the adhesive. The decrease in viscosity in this case may be due to the binding of free connections of polymer chains with microsilica molecules. The spherical shape allows them to locate in the free spaces created during the straightening and mutual relocation of the polymer chains. Importantly, however, and as proven by the studies in [52,55,56], the silica fraction can also be of great importance. As a rule, the use of nanosilica is more successful in modifying various processing properties of polymers [56,57].

In the resin, after its stabilization, the distances between the chains became larger. (The chains could not move closer to each other, as they were blocked by the distributed particles of mi-silica). The structure of the polymer became less compact, but this did not necessarily negatively affect the performance of the adhesive. This is because glue with a lower viscosity can penetrate more easily into more developed surfaces, where there are numerous cavities. When the polymer becomes more “fluid”, internal friction and mutual slippage of polymer chains occurs to a lesser extent.

By far the greatest influence on viscosity changes was the addition of carbon nanotubes. An extremely large and pronounced increase in viscosity was noted in the ER53/NT series. The reason for this is the structure of the nanotubes themselves and their behavior during sonication [50,51]. The nanotubes used in the study were three-dimensional, multilayered structures in which each carbon atom was connected to three other C atoms. This means that between these atoms, sp2 double bonds occurred with some regularity, the binding energy of which was less than that of individual sp1 bonds. During the dynamic process of sonication, they can break, resulting in tube unfolding and a significant increase in their branching surface. The structures formed in this way can enter the structures of polymer chains on the basis of mutual penetration. The compatibility of carbon atoms also greatly facilitates their bonding after the formation of chemical bonds [58,59]. This creates a secondary network structure, where the individual networks (polymeric and nanotube-derived) overlap or interpenetrate each other. As a result, the internal structure of the polymer becomes “packed”, voids disappear, and the distances between groups of atoms and molecules become small. Such changes in viscosity may favor better parametric performance of the adhesive under conditions of bonding on a relatively even surface, where cavities do not clearly reach significant values.

### 3.2. Surface Free Energy Results

As was stated earlier, the post surface free energy components responsible for the specific processes of dissolution and penetration of the adhesive into the irregularities of the concrete substrate can significantly determine the influence of the fillers used in shaping the final adhesion of the adhesive.

**Figure 8:**
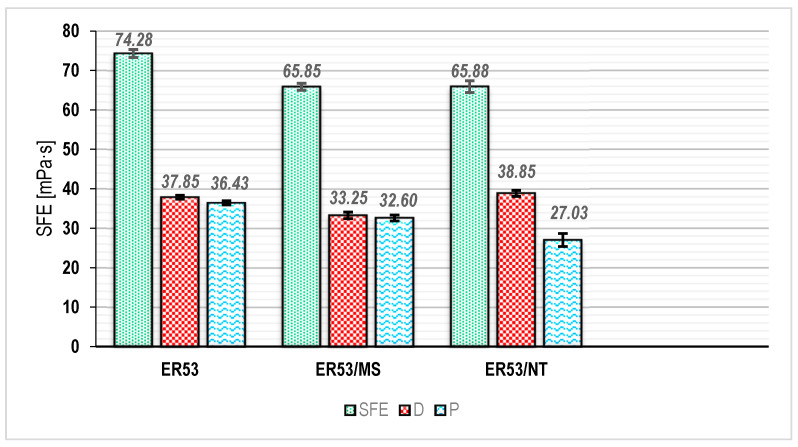
Results of surface free energy tests. D = dispersive component; P = polar component.

Figure 8 shows the obtained SFE results for each of the studied series. The surface free energy is a quantity used for a variety of phenomena. It allows the determination of the energy which must be supplied to the system of a given medium so as to produce a minimum unit area on the target substrate [1,14,15,46]. This process depends not only on the specific application of an adhesive to a substrate, for example, but also on how easily the adhesive is able to cover and fill the surface of another material. In other cases, SFE is used in evaluating the processing or hydrophobic properties of various surfaces [46,48].

Changes in the total SFE value for the series modified with filler additives were quite similar. The SFE value decreased in this case, compared with the ER53 series, by about 13.3%. It is worth noting that in the case of the unmodified series, the values of the individual components were at relatively the same level. The same was true for the values of the dispersive and polar component in the ER53/MS series. In this series, there was a 12.2% decrease in the dispersive SFE value and an 11.5% decrease in the polar value. When also considering the decrease in viscosity recorded for this series, the results of the SFE measurements suggest a greater ability of such a modified adhesive to form a uniform layer on a concrete surface. The decrease in the value of the dispersion component was clearly a result of the phenomena already described during the analysis of the viscosity results which occurred during sonication of the adhesive and filler. The lower polar component, on the other hand, allowed drawing a conclusion about the actual bonding of microsilica particles with resin molecules. These changes should be interpreted as a relatively lower capacity of the adhesive for adsorptive adhesion. At the same time, the capacity for mechanical adhesion increased, owing to the lower energy required for the adhesive to penetrate the substrate. However, the substrate itself and its energy state are also important. Lowering the value of the polar component does not preclude the formation of an adequate amount of chemical bonds between the concrete and the epoxy adhesive [57,59,60]. This is because there are media on a substrate which contain more concentrated electron clusters. Their presence and surface energy can lead to a different organization of electrons than on the surface of the resin itself and again increase adhesion. Indeed, the lower value of the polar component of SFE may also indicate the lower energy needed to “trigger” the process of adsorption adhesion formation.

The ER53/NT series showed a slightly different relationship. The polar component again showed a decrease (25.8% compared with the ER53 series adhesive and 17.1% compared with the ER53/MK series). This is related to the results shown in Figure 6. The marked increase in viscosity indicates the clearly compact and packed structure of the polymer. Most of the free electrons were confined inside the double network formed from the polymer chain and derived from the upright carbon nanotubes. Therefore, on the surface of the hardened resin, there were not as many active centers capable of forming chemical bonds with the substrate [50,58,60]. It should be remembered, however, that the adhesive was applied to the concrete while it was still in a liquid state, and thus the lower polar component may promote greater activity of the adhesive molecules in this regard. The ER53/NT series, on the other hand, showed a rather significant increase of 16.8% in the dispersion component compared with the ER53/MS series. The increased viscosity of the adhesive prepared in this way clearly limits the adhesive’s ability to spread and penetrate the substrate more deeply.

### 3.3. Results of the Grinded Surface Parameter Tests and Pull-Off Tests

**Figure 9:**
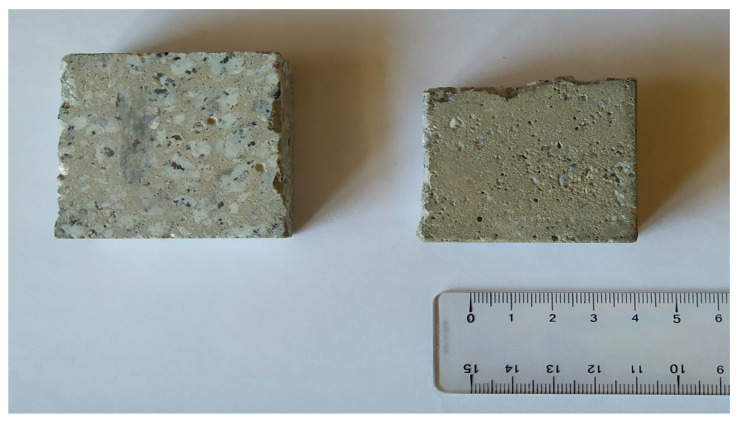
Comparison of the effects of grinding (**left**) and sandblasting (**right**) on the concrete samples.

The results obtained from the viscosity and surface free energy tests in the next stage of the study had to be referred to for determining the actual effect of the applied modifications on the adhesive condition on the concrete surface. To this end, concrete samples intended for pull-off adhesion testing of specific batches were subjected to a grinding process. This, in addition to sandblasting, brushing, and ordinary cleaning, is one of the most commonly used methods for preparing concrete substrates when reinforcing structural elements [1,15,61]. Grinding makes it possible to completely remove the damaged or degraded top layer of concrete (e.g., due to adverse weather conditions). At the same time, compared with sandblasting, which mainly leads to the removal of weak zones of a substrate without exposing the aggregate, grinding can be carried out to obtain a relatively smooth surface along with the top layer of aggregate (Figure 9).

**Figure 10:**
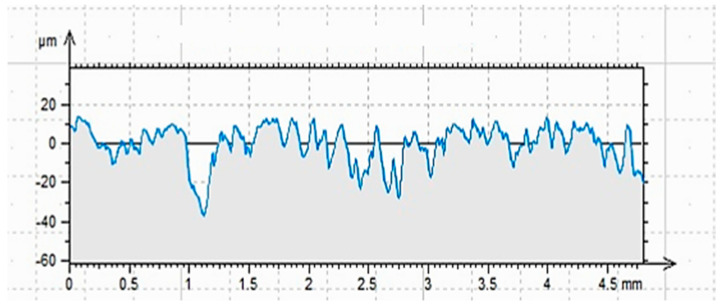
Profilogram of the ground surface G − (length = 4.80 mm; Pt = 51.0 μm; scale = 100.00 μm) [62].

**Figure 11:**
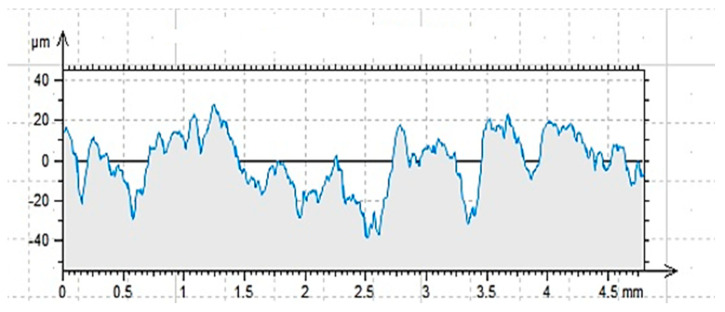
Profilogram of the sandblasted surface S − (length = 4.80 mm; Pt = 66.6 μm; scale = 100.00 μm) [62].

The results of profilometric analysis, which are also described for such a prepared surface in [62], are shown in Figure 10 and Table 4. In Figure 11, the effect of sandlasting (for comparison) is shown.

The ground surface was characterized by a relatively uniform distribution of depressions and elevations, also taking into account the overall structure of the concrete, which is never perfectly smooth, as is the case with, for example, steel [1,15,17]. The distribution of aggregate under the surface of the specimen was random but relatively regular. Most of the surface pores were abraded during grinding; only the deepest depressions remained. On a macroscopic scale, an aligned surface was observed. On the microscopic scale, the effect was clearly visible. The average height of the elements of the height profile (Rc) was more than double that of the profile of a cleaned surface, for example [62]. The obtained values for the depressions and elevations (R_p_ and R_v_, respectively), as well as the width of the grooves R_sm_, are the basic parameters for determining the ability of an adhesive to wet a surface prepared in this way. On the other hand, the value of the parameter (R_kn_ ≈ 3) indicates a normal distribution of values for the profile properties [15].

The exact results of adhesion of the adhesives, with the help of which fragments of CFRP tape were glued to the prepared concrete, are presented in Table 5. The pull-off method made it possible to quickly determine the adhesion [63]. Assuming axial application of the pull-off force to the specimen, the main type of stress was normal stresses, which next to shear stresses are the main type of stresses occurring in structural member reinforcement systems using CFRP tapes.

The final adhesion of the unmodified adhesive and modified series was the result of several factors. It should be emphasized that the results obtained were not solely due to changes in the most commonly described mechanical adhesion. The ER53/MS series showed a 15.9% increase in pull-off adhesion, while the ER53/NT series showed a 38.2% increase over the ER53 series and a 19.2% increase over the ER53/MS series. Since all of the specimens were bonded to a concrete surface prepared in the same way, this suggests changes in adhesion not only mechanically, but more importantly, adsorptively. These changes, in turn, are clearly dependent on the type of filler added, as was the case with the viscosity and surface free energy results [58,59]. It is possible that in the situation of applying an adhesive to a concrete substrate, which according to Lewis’ theory of acids and bases is an electron donor, there was a transition of electrons accumulated on the surface to the adhesive structure. This was all the more possible due to the presence and exposure of granite aggregate grains in the concrete as a result of grinding, the predominant chemical composition of which is silica. The chemical compatibility of the filler (microsilica), resin, and substrate allowed the formation of chemical bonds. Their durability was sufficient to increase the adhesion strength of the adhesive. The much greater increase in adhesion of the ER53/NT series of adhesives was due to the higher viscosity, lower surface energy, and most likely the greater amount of chemical bonding between the adhesive and the substrate. As was already described, the developed carbon nanotubes and polymer network became two interpenetrating structures. Therefore, the number of molecules which participated in electron exchange between the substrate and the adhesive also increased. The increase in adhesion also suggests higher energy and durability for such bonds. In an epoxy adhesive, there are methyl groups, phenolic groups, epoxides, epitoxides, and other molecules which are components of polymer chains [24]. The effects of sonication and filler additives caused specific interactions between individual molecules, as described when analyzing the viscosity and SFE changes. The ER53/MS adhesive, due to changes in viscosity and SFE, obtained a greater ability to penetrate a rough surface with a relatively regular pattern of depressions and elevations. On the other hand, the ER53/NT adhesive obtained the ability to adhere to the surface more effectively and form joints according to the principles of physicochemistry [48].

The viscosity, SFE, and pull-off adhesion results obtained indicate that such selected fillers can be used with other epoxy adhesives. Further optimization of the adhesive properties can be achieved by using a different amount of fillers, using a different sonication time, and choosing other adhesives, including those which have a ready-made base filler in their composition, such as in the form of quartz flour.

### 3.4. SEM Analysis

**Figure 12:**
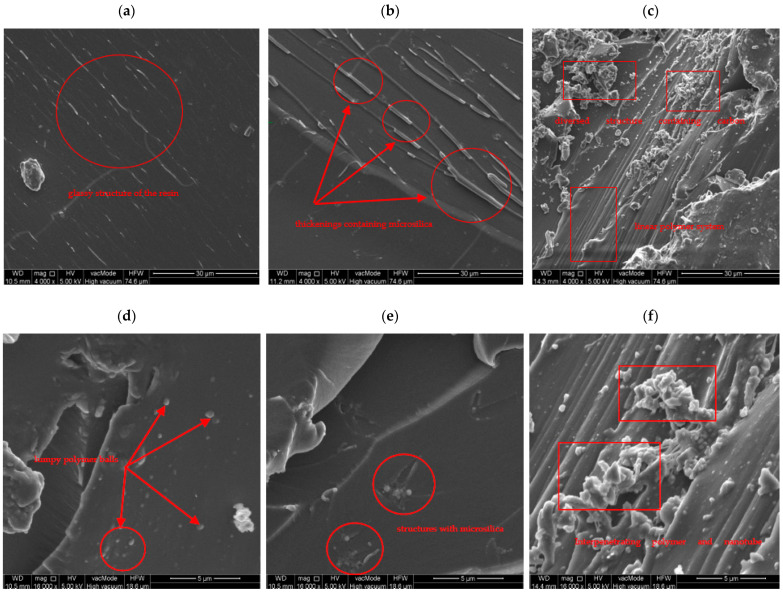
SEM pictures of modified resins: (**a**–**c**) 4.000× magnification, (**d**–**f**) 16.000× magnification, (**a**,**d**) ER53 series, (**b**,**e**) ER53/MS series, and (**c**,**f**) ER53/NT series.

The results obtained from testing the viscosity, SFE, and adhesion of the analyzed series of adhesives to concrete showed the effectiveness of the adopted modifications. However, they did not allow determining the actual changes in the microstructure of the adhesives. In order answer this question, an SEM analysis of the fragments of the samples taken from their surfaces, which were directly adjacent to the rough substrate under bonding conditions, was carried out. The images taken are shown in Figure 12. Similar analyses of epoxy adhesives with additives of microsilica and carbon nanotubes are described in [64,65,66].

The 4000 and 16,000 fold magnification photos show some important differences in the surface textures of the resins. In the case of the ER53 series, it was relatively smooth, glassy, and ordered, which is typical of epoxy resins (Figure 12a). A certain orderly, linear course of the structure is visible, which characterizes systems based on the chain structure. At higher magnifications, lumpy microstructures are visible, which indicate the coiling of polymer chains at their ends (Figure 12d). This happens when the polymer reverts to its base form. The clumped formations are most energetically efficient when the adhesive is not involved in binding to the substrate. Nonetheless, when the adhesive is applied to the substrate, they can re-form, aiming to form a bond. In the case of the ER53/MS series (Figure 12b), the glassy form of the resin is also visible, but the linearly oriented structures became thickened. This indicates some changes which occurred in the microstructure under the influence of the addition of microsilica. It is likely that the visible thickening was the result of bonding of microsilica particles with free polymer ends containing functional groups. In such cases, the van der Waals bonds were the most common chemical bonds. The occurrence of such structures resulted in the formation of fewer glomerular ends of polymers in comparison with unmodified adhesive (Figure 12e). The structure formed in this way also bound more easily to the substrate, as activation of electron flow occurred faster. When also considering the previous viscosity results, it can be concluded that locating the microsilica molecules between the structures of the polymer chains effectively prevented their proximity to each other and resulted in a decrease in viscosity. This in turn resulted in easier penetration of the adhesive into the irregularities of the concrete substrate.

On the other hand, the structures in the SEM images taken for the ER53/NT series were decidedly different. Two interpenetrating networks, polymeric and nanotube, resulted in highly differentiated structures. The glassy form of the resin with some linear arrangement of the polymer is still visible (Figure 12c). However, numerous kinks, lumps, branches, and free ends are also visible on the surface. These are quite pronounced, translating into a varied topography for the adhesive surface in contact with the concrete. As a result, numerous centers were formed which contained electrons capable of forming permanent chemical bonds with the substrate. The structure itself also showed a clearly compact form, which translated into a significant increase in the viscosity of the adhesive.

## 4. Conclusions

As a summary of the research conducted, several important issues should be highlighted. The main goal of trying to increase the adhesion of epoxy resin to concrete (ground surface) was achieved. In the case of modification with the addition of microsilica, this adhesion increased by about 16%, and in the case of the series with the addition of multilayer carbon nanotubes, it increased by more than 38%. It was possible to observe significant changes in viscosity and the values of the particular components (dispersive and polar) for the surface free energy for the final increase in adhesion. Analysis of SEM images indicated significant differences in the localization of molecules of added fillers in the main structure of the resin. The use of sonication as a method for mixing resin and fillers is highly beneficial. This is due to the phenomenon of cavitation accompanying sonication and the thermodynamic changes occurring in the resin.

Implementation of the described modifications is not difficult from a technological point of view. However, the extremely high precision of the work performed is important. In the event of possible use of the described methods under the conditions of the actual execution of reinforcement (e.g., for a reinforced concrete element), special attention should be paid to the conditions for glue preparation.

The results obtained are valuable from the point of view of the technology of strengthening concrete and reinforced concrete structures using CFRP composites. Further development of the work in this direction can be directed toward the adoption of other types of fillers and studying the effects of the described modifications in relation to other FRP composites and with the adoption of other parameters of the sonication itself.

## Figures and Tables

**Table 1:** Properties of the resin used in the tests.

Resin	Epidian 53 (ER53)
Form	brown, dense liquid
Flashpoint (°C)	75
Gelation time (min)	60
Epoxy number (mol/100 g)	0.4
Density (22 °C) (g/cm^3^)	1.12–1.15
Viscosity (22 °C) (Pa·s)	0.9–1.5
Solubility	ketones, esters, alcohols
Chemical resistance	tap water, sodium hydroxide, hydrochloric acid, concentrated hydrochloric acid, sulfuric acid, nitric acid, acetic acid, xylene, ethanol

**Table 2:** Properties of fillers used in tests with MS and NT as symbols used in the research.

**Filler**	Microsilica (MS)	carbon nanotubes (NT)
**Form**	spherical particles	multilayer nanotubes
**Density (22 °C) (g/cm^3^)**	2.2	1.3–1.4
**Dimension**	0.1 µm (diameter)	9.5 nm (diameter), 1.5 µm (length)
**Specific surface area (m^2^/g)**	20	250–300

**Table 3:** Recipes and series used in research.

Series	Resin Type	Type of Additive or Modification	Amount of Filler (%)	Amount of Hardener (%)
ER53	epoxy	-	-	10
ER53/MS		sonication + microsilica	0.5	10
ER53/NR		sonication + carbon nanotubes	0.1	10

**Table 4:** Roughness profile parameters for individual surfaces (µm). G = grinded surface [62].

Surface	R_p_	R_v_	R_z_	R_c_	R_t_	R_a_	R_q_	R_sm_	R_sk_	R_ku_
**G**	12.60	17.10	29.70	16.00	45.00	5.66	6.96	0.15	−0.42	2.70

**Table 5:** Results of pull-off measurements for tested glue.

Series	Force (kN)	Pull-Off Stress (MPa)	Coefficient of Variation (%)
ER53	3.81	5.16	3.0
ER53/MS	4.39	5.98	3.0
ER53/NT	5.35	7.13	2.0

## Data Availability

Data are contained within the article.
